# Evaluation of Signaling Pathways Profiling in Human Dermal Endothelial Cells Treated by Snake Venom Cysteine-Rich Secretory Proteins (svCRiSPs) from North American Snakes Using Reverse Phase Protein Array (RPPA)

**DOI:** 10.3390/toxins13090613

**Published:** 2021-08-31

**Authors:** Montamas Suntravat, Oscar Sanchez, Armando Reyes, Abcde Cirilo, Jack S. Ocheltree, Jacob A. Galan, Emelyn Salazar, Peter Davies, Elda E. Sanchez

**Affiliations:** 1 National Natural Toxins Research Center (NNTRC), Texas A&M University-Kingsville, MSC 224, 975 West Avenue B, Kingsville, TX 78363, USA; oscar.sanchez@tamuk.edu (O.S.); armando.reyes@students.tamuk.edu (A.R.); abcde.cirilo@students.tamuk.edu (A.C.); jack.ocheltree@students.tamuk.edu (J.S.O.); jacob.galan@tamuk.edu (J.A.G.); emelyn.salazarcastillo@tamuk.edu (E.S.); elda.sanchez@tamuk.edu (E.E.S.); 2 Department of Chemistry, Texas A&M University-Kingsville, MSC 161, Kingsville, TX 78363, USA; 3 Institute of Biosciences and Technology, Texas A&M University, Houston, TX 77843, USA; pdavies@tamu.edu

**Keywords:** North American snakes, snake venom cysteine-rich secretory proteins (svCRiSPs), endothelial permeability, reverse phase protein arrays (RPPA), signaling pathway

## Abstract

Cysteine-Rich Secretory Proteins (CRiSPs) are typically found in many snake venoms; however, the role that these toxins play in the pathophysiology of snakebites is still unclear. Herein, we compared the effects of snake venom CRiSPs (svCRiSPs) from the most medically important species of North American snakes on endothelial cell permeability and vascular permeability. We used reverse phase protein array (RPPA) to identify key signaling molecules on human dermal lymphatic (HDLECs) and blood (HDBECs) endothelial cells treated with svCRiSPs. The results showed that Css-CRiSP isolated from *Crotalus scutulatus scutulatus* and App-CRiSP from *Agkistrodon piscivorus piscivorus* are the most potent causes of increase vascular and endothelial permeability in comparison with other svCRiSPs used in this study. We examined the protein expression levels and their activated phosphorylation states in HDLECs and HDBECs induced by App-CRiSP and Css-CRiSP using RPPA. Interestingly, both App-CRiSP and Css-CRiSP induced caveolin-1 expression in HDBECs. We also found that stimulating HDBECs with Css-CRiSP and App-CRiSP significantly induced the phosphorylation of mTOR and Src, respectively. In HDLECs, Css-CRiSP significantly downregulated the expression of N-Cadherin and phospholipase C-gamma, while App-CRiSP significantly enhanced Akt and JNK phosphorylation. These results suggest that the increased endothelial permeability in HDLECs and HDBECs by Css-CRiSP and App-CRiSP may occur through different pathways.

## 1. Introduction

Snakebites have been recognized as one of the most important neglected tropical diseases by the World Health Organization (WHO). They are a major human health problem, leading to an estimated 138,000 deaths annually and severe injury to more than 2.7 million people [1]. Despite its importance as a global health issue, the pathophysiology of snakebites remains unclear. Snake venoms are comprised of various proteins and peptides. Among them, snake venom cysteine-rich secretory proteins (svCRiSPs) are ubiquitous venom components in many species of snakes [1,2], including Elapidae (Elapinae and Hydrophiinae), Viperidae (Viperinae and Crotalinae) [3], and Colubridae [4]. Increasing evidence and characterization on the structural and biological activities of svCRiSPs have been from those purified from the venoms of Asian and Australian snakes, largely in part because of the snake venom research in those geographic regions.

Numerous svCRiSPs have been reported to block ion channel activities such as L-type Ca^2+^ and/or K^+^ channels [5,6]. Two svCRiSPs, natrin, a well-characterized svCRiSP isolated from *Naja atra* venom, and ES-CRiSP from the venom of *Echis carinatus sochureki*, have been shown to have diverse effects on endothelial cell functions [7,8]. Natrin was able to induce the expression of adhesion molecules and activate the mitogen-activated protein kinases (MAPKs) including extracellular signal-regulated kinases (ERK), C-Jun N-terminal kinase (JNK), and p38 MAPK in human umbilical vein endothelial cells (HUVEC) [7]. While ES-CRiSP has been found to inhibit the proliferation of HUVEC and glioma human microvascular endothelial cells (gHMVEC), it has no effect on MAPKs including p38 kinase and stress-activated protein kinase (SAPK)/JNK but can inhibit the activation of ERK1/2 induced by vascular endothelial growth factor (VEGF) in HUVEC cells [8]. Though the identification of key signaling molecules has been identified, the molecular mechanisms and molecular targets of most svCRiSPs are still unknown. Additionally, few studies have focused on the biological functions and cellular signaling of svCRiSPs in the venoms of North American snakes, especially cottonmouths and copperheads (Genus *Agkistrodon*) and rattlesnakes (Genus *Crotalus* and *Sistrurus*). Adade et al. [9] reported that crovirin, a svCRiSP isolated from the North American Prairie rattlesnake (*Crotalus viridis viridis*) venom, exhibited anti-protozoal activity.

Our previous work reported that Hellerin, a newly discovered svCRiSP purified from *C. oreganus helleri* venom, increases vascular permeability in vivo and endothelial permeability in vitro, and also targets the lymphatic cells [10]. Here, we extended the characterization of the comparative vascular and endothelial permeabilities of multiple svCRiSPs isolated from the venoms of North American snakes and identification of key signaling pathways in human endothelial cells induced by svCRiSPs using reverse phase protein array (RPPA) to improve the understanding of the cellular and molecular mechanisms of the toxins and their role in the complex pathophysiological responses following snakebite envenoming.

This work, for the first time, to our knowledge, identifies several adhesion membrane proteins and signaling molecules as candidate regulators of endothelial monolayer permeability in HDBECs and HDLECs induced by crotaline CRiSPs.

## 2. Results and Discussion

### 2.1. Venomic Analysis and Purification of svCRiSPs

**Figure 1:**
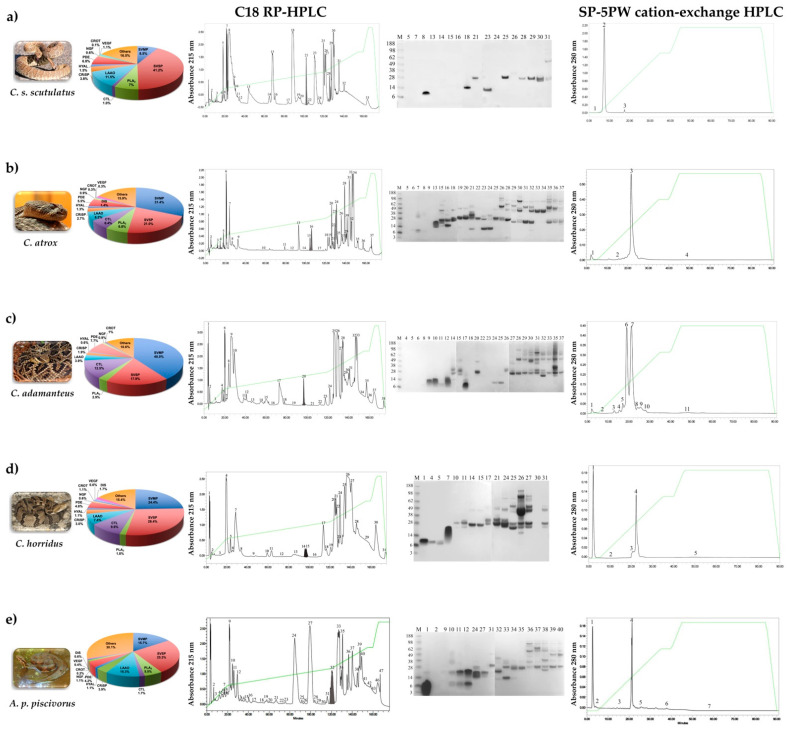

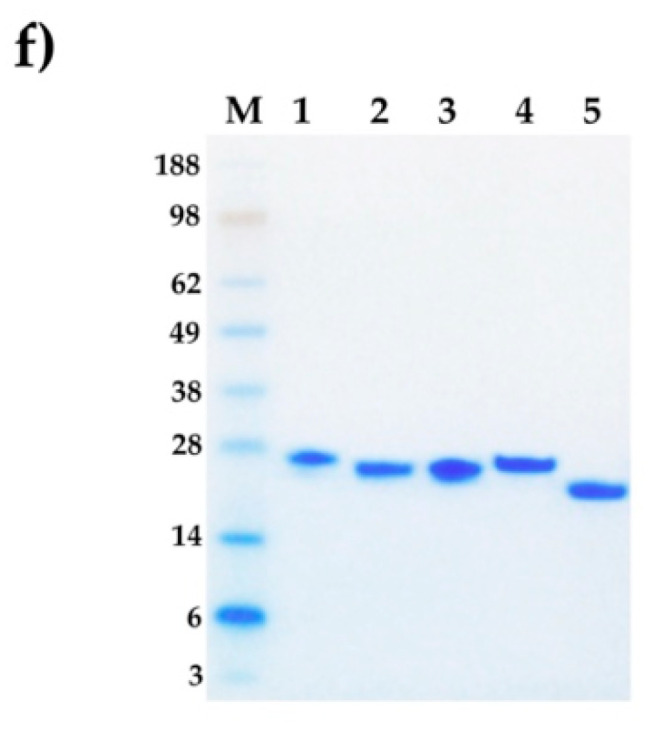
Venomic profiles and purification of svCRiSPs from the venom of five crotaline snakes. *C. s. scutulatus* (**a**), *C. atrox* (**b**), *C. adamanteus* (**c**), *C. horridus* (**d**), and *A. p. piscivorus* (**e**) venoms were sequentially fractionated by C18 RP-HPLC followed by SP-5PW cation-exchange HPLC. The grey-shaded areas indicate the fractions containing svCRiSPs, which were confirmed by N-terminal sequencing. Pie charts show the relative abundance of venom toxin families in each venom from five individuals. SVSP: Snake venom serine protease, SVMP: Snake venom metalloproteinase, PLA_2_: Phospholipase A_2_, CRiSP: Cysteine-rich secretory protein, LAAO: L-amino acid oxidase, CTL: C-type lectin, HYAL: Hyaluronidase, NGF: Nerve growth factor, PDE: Phosphodiesterase, VEGF: Vascular endothelial growth factor, and CLP: Crotamine-like peptide. All fractions were collected manually and determined by SDS-PAGE under non-reducing conditions. Elution profiles of the venoms fractionated in several peaks represented by their number. (**f**) SDS-PAGE of purified svCRiSPs. Purified crotaline CRiSPs (5 μg) were applied on 4–12% (*w*/*v*) Bis-Tris gel in non-reducing conditions. M: Molecular mass marker (SeeBlue Plus2 Markers, Invitrogen™). Lane 1: Css-CRiSP; lane 2: Catrox-CRiSP; lane 3: Cada-CRiSP; lane 4: Chor-CRiSP; lane 5: App-CRiSP.

Snake venoms are highly complex cocktails of various bioactive compounds and are a resource for uncovering new svCRiSPs that can be used to explore molecular mechanisms of these toxins underlying blood and lymphatic endothelial permeability and vascular function. To determine the presence of CRiSP in the venoms used in this study, we evaluated the venomic profiles of the *C. s. scutulatus*, *C. atrox*, *C. horridus*, *C. adamanteus*, and *A. p. piscivorus* using LC-MS/MS analysis. Our analysis determined the venom components among all individual snakes along with their estimated relative abundances. The proteins were grouped into 12 protein families including snake venom serine proteases (SVSPs), snake venom metalloproteinases (SVMPs), phospholipases A_2_ (PLA_2_s), CRiSPs, L-amino acid oxidases (LAAOs), C-type lectins (CTLs), hyaluronidases (HYALs), nerve growth factors (NGFs), phosphodiesterases (PDEs), VEGFs, and crotamine-like peptides (CLPs). The peptide sequences and data of mass spectrometry are available in Supplementary Tables S1–S5. As shown in Figure 1a–e, all the venom samples contained CRiSPs, which accounted for 1.9–4% of the total venom proteomes. This finding was in agreement with the previous proteomic and transcriptomic studies for these snakes [11,12,13,14].

svCRiSPs (Css-CRiSP, Catrox-CRiSP, Cada-CRiSP, Chor-CRiSP, and App-CRiSP) were isolated from five crotaline snake venoms using two chromatographic protocols including a C18 reverse phase (RP) high-performance liquid chromatography (HPLC) and subsequent cation exchange HPLC (Figure 1a–e). The SDS-PAGE analysis of purified svCRiSPs in non-reducing conditions revealed the protein bands with molecular mass of about 24–26 kDa (Figure 1f). Using N-terminal amino acid sequencing, these purified proteins were validated as svCRiSPs.

### 2.2. Full Amino Acid Sequences of svCRiSPs

The protein bands of svCRiSPs were excised from SDS-PAGE, followed by tryptic digestion. The tryptic digested peptides were analyzed by LC-MS/MS mass spectrometry. All five svCRiSPs were validated by N-terminal sequencing together with mass spectrometry (Table 1). The partially overlapping and redundant sets of peptides were included in Supplementary Table S6.

**Figure 2:**
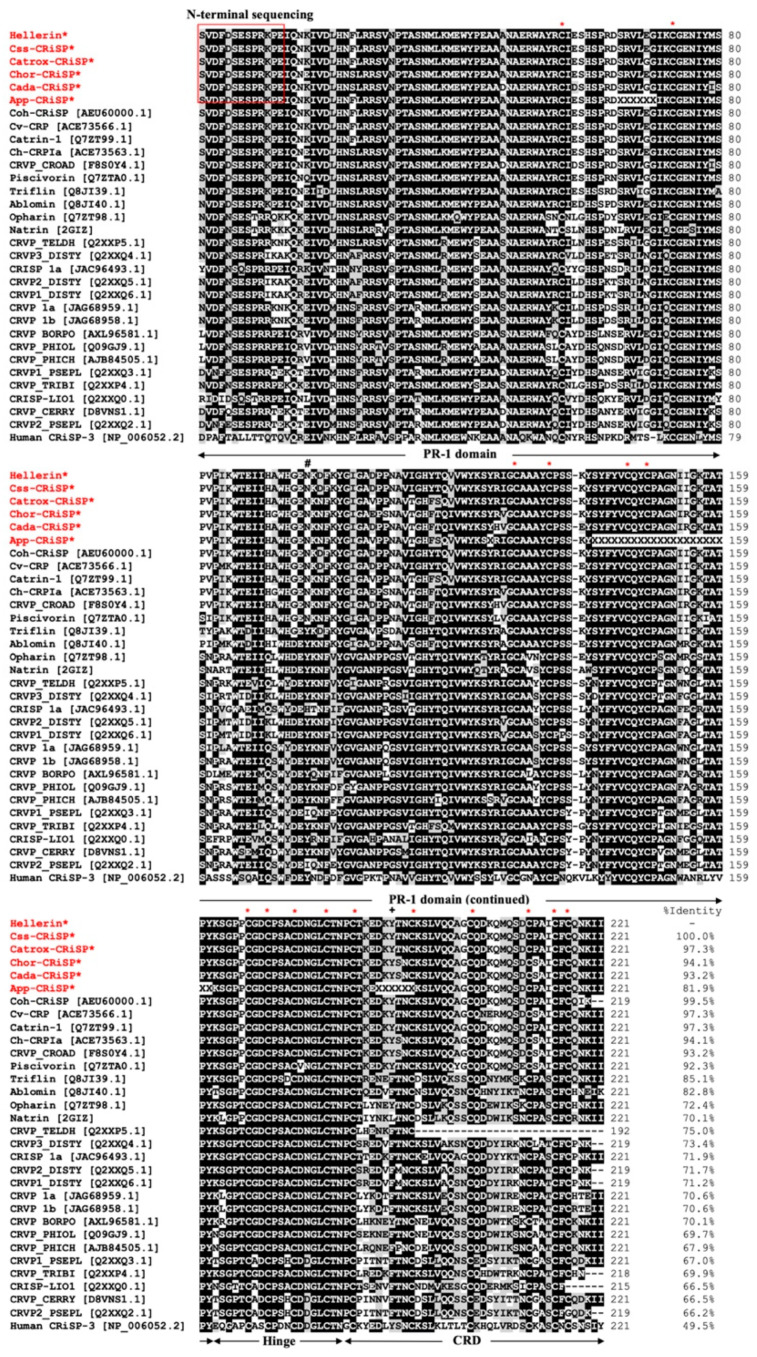
Multiple sequence alignments of svCRiSPs with homologous proteins from other snake species using ClustalW. The asterisk (*) indicates the highly conserved cysteine residues. The “#” and the “+” indicate two different amino acid residues at the position 97 (within the PR-1 domain) and 189 (within the CRD domain) between the North American species and the Asian species.

**Figure 3:**
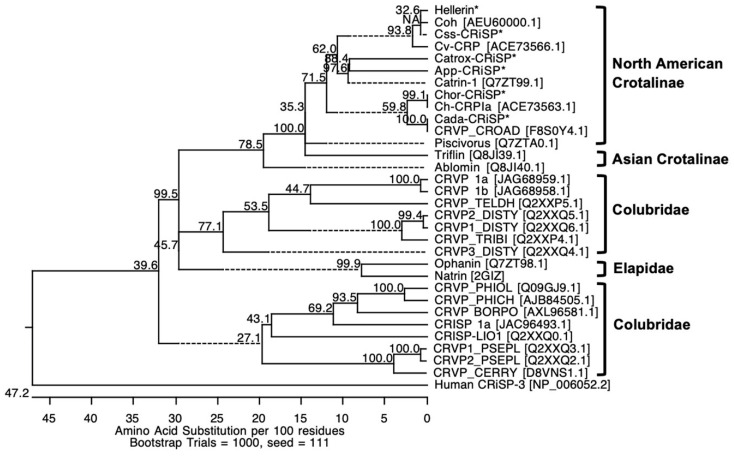
Phylogenetic analysis of crotaline CRiSPs based on their amino acid sequences from N-terminal sequencing and LC-MS/MS. Crotaline CRiSPs are indicated by an asterisk (*). Human CRiSP-3 (NP_006052.2) was included as an outgroup. The phylogenetic tree was generated by the neighbor-joining method. The bootstrap test was performed using 1000 replications. Numbers on nodes are the bootstrap confidence values.

We compared the primary amino acid sequences of crotaline CRiSPs with other svCRiSPs in a database. The amino acid sequences of all svCRiSPs contain 16 conserved cysteine residues (Figure 2). These crotaline CRiSPs share high levels of sequence similarity among them with only few different amino acid residues. Interestingly, Css-CRiSP had 100% identity with Hellerin. Crotaline CRiSPs had 80–97% identity to each other, but less identity to elapid CRiSPs (70–72%), and colubrid CRiSPs (66–75%) (Figure 2). We constructed the phylogenetic tree based on full-length amino acid sequences of svCRiSPs (Figure 3). We grouped snake subfamilies or families as various clades. CRiSP-3 from human was used as an outgroup. We found that North American crotaline CRiSPs were grouped with each other. svCRiSPs isolated from Asian snakes (triflin isolated from the *Trimeresurus flavoviridis* and ablomin from *Agkistrodon blomhoffi*) were clustered in the same group.

We discovered two different amino acid residues at positions 97 and 189 between two different crotaline-clades of svCRiSPs, which are indicated by the “#” and “+”, respectively (Figure 2). These differences result in Y97/F189 in the Asian CRiSPs and N97/Y189 in the North American CRiSPs. We also found 15 residues different among crotaline CRiSPs within the pathogenesis-related 1 (PR-1) domain and 2 residues different within the cysteine-rich domain (CRD) domain. The structural characterization of svCRiSPs demonstrated that the PR-1 domain is implicated in the ligand-target binding and the CRD domain governs binding selectivity to their targets, in particular ion channels [5,15]. Small variations in the primary amino acid sequences could be responsible for their molecular target affinity and avidity resulting in distinct biological functions.

### 2.3. Measurement of Vascular Permeability and Endothelial Permeability

**Figure 4:**
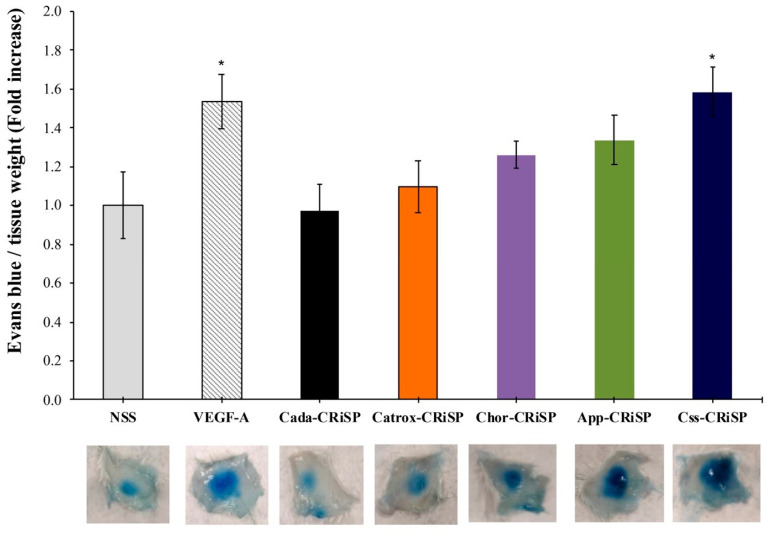
Effects of svCRiSPs on vascular permeability using Miles assay in vivo. Quantitative analysis of extravasation of intravascular dye (Evans blue) in the skin samples after subcutaneous administration of svCRiSPs (170 ng), VEGF-A (170 ng), or saline for 30 min. The data represent vascular permeability expressed as a fold increase relative to saline control. The results are presented by mean + SE (n = 5). Statistical significance determined by Student’s *t*-test (* *p* < 0.05).

Increased vascular permeability causes vascular leakage and edema and is commonly found in many pathological conditions such as severe trauma, inflammatory diseases, burns and drug toxicity, as well as snake envenoming. We have demonstrated that our first CRiSP, Hellerin, rapidly increases vascular permeability in vivo and blood and lymphatic endothelial permeability in vitro [10]. In order to identify the biological activities of svCRiSPs isolated from different species of snakes, we characterized the effects of all svCRiSPs on vascular permeability, the cytotoxicity, and endothelial permeability in human dermal endothelial cells (HDLECs) and human dermal blood endothelial cells (HDBECs). Vascular permeability was performed in mice using Miles assay. The leakage of Evans blue dye in mice induced by Css-CRiSP (170 ng/mouse) significantly increased by 59% compared with the negative control group (saline) (Figure 4). This activity was comparable to treatment with VEGF-A (54%), a known enhancer of vascular permeability. Chor-CRiSP and App-CRiSP were able to induce the vascular permeability by 26% and 33%, respectively. In contrast, the extravasation of Evans blue dye was less pronounced in mice treated with Catrox-CRiSP and Cada-CRiSP.

To confirm that crotaline svCRiSPs enhanced permeability was not due to cytotoxic activity, all five svCRiSPs were initially tested for cytotoxicity on HDLECs and HDBECs. There was no evidence of cytotoxicity in either HDBECs or HDLECs as indicated in an absence of detectable morphological changes or cell viability as measured by a Cell Titer-Blue assay.

**Figure 5:**
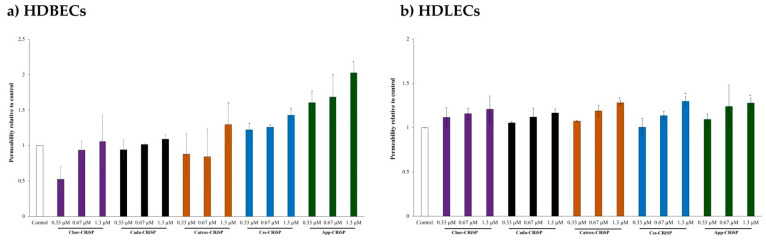
Endothelial permeability of HDBECs (**a**) and HDLECs (**b**) induced by svCRiSPs. Confluent HDBECs and HDLECs grown on gelatin-coated transwells were serum starved for 3 h before stimulation with svCRiSPs at different concentrations for 1 h. FITC-BSA was added to the top compartment of transwells for 30 min and subsequently measured the fluorescent intensity in the bottom compartments. Values are expressed as fold-change compared to control and presented as mean ± SD from three independent experiments (n = 3). Statistical significance determined by Student’s *t*-test (* *p* < 0.05).

To further examine the effect of svCRiSPs on the permeability of blood and lymphatic endothelial cells, we performed an in vitro endothelial permeability assay. As shown in Figure 5, we observed that Catrox-CRiSP, Css-CRiSP, and App-CRiSP significantly increased the permeability in HDBEC after 1 h administration in a dose-dependent manner. While at 1.3 µM, App-CRiSP, Catrox-CRiSP, and Css-CRiSP but not Chor-CRiSP and Cada-CRiSP, induced significant increases in HDLECs permeability by 27.8%, 28.2%, and 30%, respectively, as compared with that in PBS-treated cells. These results confirm that Css-CRiSP, App-CRiSP, and Catrox-CRiSP induced acute changes in both HDBEC and HDLEC cells barrier function, while Cada-CRiSP and Chor-CRiSP had no detectable activity in both blood and lymphatic endothelial cells. By using both in vivo assay of vascular permeability and in vitro assay on HDBECs and HDLECs permeability, our data clearly show that svCRiSPs have direct effects on endothelial cell barrier function. App-CRiSP and Css-CRiSP were the most potent inducers of vascular and endothelial permeability in comparison with other crotaline CRiSPs.

### 2.4. Reverse Phase Protein Array (RPPA)

**Figure 6:**
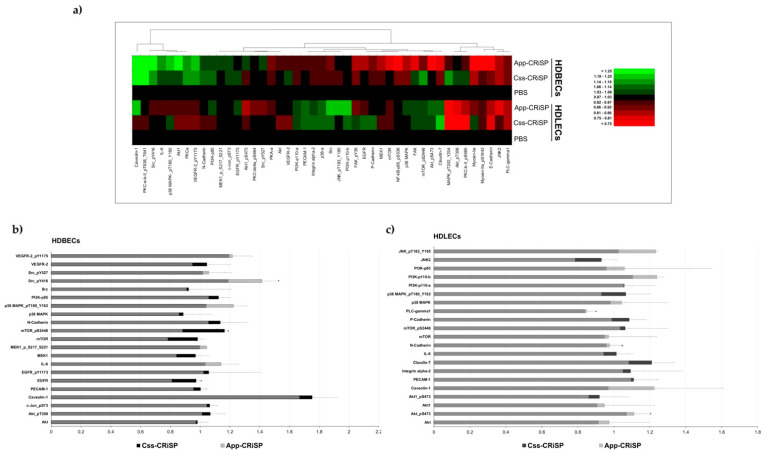
The effect of Css-CRiSP and App-CRiSP on signaling pathways in HDBEC and HDLEC cells. HDBEC and HDLEC cells were treated for 30 min with Css-CRiSP and App-CRiSP (1 µM) or PBS (control) and protein lysates were analyzed by RPPA. (**a**) Heat map of protein expression profiles based on RPPA. Values are expressed as log2 fold difference from the control (PBS) samples. Green color represents high protein expression, and red indicates low protein expression. Fold change of the selected proteins in HDBECs (**b**) and HDLECs (**c**) compared to the PBS control. Data are presented as mean ± SD from two independent experiments (n = 2). Statistical significance determined by Student’s *t*-test (* *p* < 0.05).

RPPA is a high-throughput approach for quantitative analysis of the protein expression levels and post-translational modifications (e.g., phosphorylation) of many proteins signaling networks [16]. We employed RPPA to profile and identify the potential signaling pathways that are responsible for endothelial permeability induced by Css-CRiSP and App-CRiSP. HDBECs and HDLECs were treated with control (PBS), Css-CRiSP (1 μM), or App-CRiSP (1 μM) for 30 min and cell lysates were subjected to RPPA. A total of 439 validated antibodies were used to probe the RPPA and are available in the RPPA Core Facility at MD Anderson. These total/phospho-specific antibodies specifically detect proteins involved in several intracellular signaling pathways, such as MAPK pathway, receptor tyrosine kinases, phosphatidylinositol 3-kinase (PI3K)-Akt signaling, AMP-activated protein kinase (AMPK) signaling, mTOR pathway, protein kinase C (PKC) signaling, cell adhesion, cell migration, cell stress, cell cycle, cell death, DNA repair, and immune response. A heat map was used to graphically show the fold change in protein expression relative to the control for 45 proteins associated with cell permeability, in response to treatment by Css-CRiSP and App-CRiSP in HDLECs and HDBECs (Figure 6a, Supplementary Table S7).

Endothelial cell–cell junctions play an important role to maintain a highly restrictive barrier between endothelial cells, regulate vascular integrity and leukocyte extravasation by a dynamic balance between cell junction proteins and many signaling molecules. It has been shown that increased endothelial permeability is associated with the alteration of cell-cell junctions, the phosphorylation of endothelial tight junction membrane proteins, transcytosis of macromolecules through the microvascular endothelial barrier, and activating intracellular signaling events [17,18]. Interactions between the structural components of endothelial junctions play a crucial role for supporting a proper barrier function. There are a number of transmembrane receptors with highly specialized functions, such as junctional adhesion molecules (JAMs), occludin, claudin family proteins, and cadherins that are involved in cell-to-cell junctions and intracellular signaling transduction via multiple distinct mechanisms [19,20,21].

RPPA-based quantification of protein expression and phosphorylation in starved, svCRiSPs-treated cells revealed that adhesion and intracellular signaling molecules were altered in Css-CRiSP and App-CRiSP samples, compared with untreated cells (Figure 6b,c). We observed that both Css-CRiSP and App-CRiSP induced the expression of caveolin-1 (cav-1) in HDBECs (Figure 6b); however, the protein levels did not change significantly when compared with the untreated control.

Cav-1 is an integral membrane protein with a molecular weight of about 21–24 kDa and it plays an important role in maintaining the formation of plasma membrane caveolae. Cav-1 in caveolae has been shown to be involved in multiple cellular functions, including potocytosis, transcytosis, cell proliferation and differentiation, cellular signal transduction, and vascular permeability [22,23,24,25]. Enhanced phosphorylation and overexpression of Cav-1 in endothelial cells increase transcellular albumin permeability by lipopolysaccharide [26]. Cav-1-deficient endothelial cells were able to reverse the increase in endothelial permeability induced by hypoxic trophoblast conditioned medium [27]. In this study, we showed that both Css-CRiSP and App-CRiSP upregulate Cav-1 expression, suggesting a possible role in the regulation of endothelial permeability in HDBECs; however, additional studies are needed to confirm these observations.

This screening also suggested that several candidate proteins belonging to the adhesion membrane proteins and Akt/mTOR and Src/JNK pathways were significantly modified. We observed that Css-CRiSP significantly downregulated phospholipase C-gamma (PLC-gamma) and neural cadherin (N-Cadherin) in HDLECs, whereas mTOR-pS2448 was significantly upregulated in HDBECs (Figure 6b,c and Supplementary Table S7).

Cadherins are a member of calcium-dependent adhesion membrane proteins and are known to regulate junction stability and endothelial permeability [28]. Two major endothelial cadherins are neural cadherin (N-Cadherin) and vascular endothelial cadherin (VE-Cadherin). N-cadherin is expressed along with VE-cadherin in endothelial cells and has been found to be essential for the development of embryonic angiogenesis and for maintaining vascular integrity [29]. Kruse et al. [30] showed that lack of N-cadherin in either pericytes or endothelial cells results in increasing junctional permeability in the brain and lung, which leads to a decrease of VE-Cadherin accumulation at the adherens junctions.

Akt-pS473 and Src-pY416 were significantly upregulated upon App-CRiSP treatment in HDLECs and HDBECs, respectively (Figure 6b,c and Supplementary Table S7). Furthermore, calculations of phospho/total protein ratios (>1.5-fold induction or regression) of the investigated proteins revealed that App-CRiSP treatment resulted in upregulation of JNK-pT183-Y185 in HDLECs as well as upregulation of Src-pY416 in HDBECs (Supplementary Table S8).

Several studies have demonstrated that increased vascular barrier leakage with different types of pathophysiological stimuli is involved in the deregulation of several pathways such as PKC [31,32], PI3K [33], myosin light chain kinase (MLCK) [34], and p38 MAPK [32,35]. Alteration of key proteins in these signaling pathways can lead to the change of tight junctional proteins expression and disruption in their integrity and assembly, contributing to increased permeability [36]. For instance, VEGF, a well-known activator of vascular permeability, plays a central role in vascular permeability [37,38]. VEGF has been reported to induce endothelial permeability via Src, ERK, JNK, PI3K/Akt-dependent phosphorylation, PLC-dependent calcium release, and VE-Cadherin internalization [39,40,41,42,43]. In our observations, App-CRiSP also induced an increase in phosphorylated JNK in HDLECs. Likewise, Wang et al. [7] showed acute phosphorylation of JNK in HUVECs cells treated with natrin.

Our study provides observations that suggest App-CRiSP and Css-CRiSP enhance the endothelial permeability in HDLECs and HDBECs through distinct pathways. RPPA is a valuable platform to evaluate key signaling pathways promoting endothelial dysfunction in response to svCRiSP activation. However, the success of RPPA depends on the availability and quality of antibodies used. These interesting observations from the RPPA platform supports the need for further in-depth investigation.

## 3. Conclusions

These results will provide a pathomechanistic answer to the question of whether svCRiSPs induce different adhesion molecules and cell signaling pathways leading to cell permeability, which contributes to the complex physiological process in snakebites. This study opens new opportunities for the discovery of novel molecular targets of svCRiSPs. It can be exploited to extend knowledge gained from these studies to other svCRiSPs and provide insights into the general effects of svCRiSPs on vascular function and the possible role that these effects may play in the pathophysiology of snakebite.

## 4. Materials and Methods

### 4.1. Snake Venoms

Snakes are housed in a single captivity in the serpentarium at the National Natural Toxins Research Center (NNTRC) in Texas A&M University-Kingsville (TAMUK). Lyophilized crude venoms of *C. s. scutulatus* (Mohave rattlesnake), *C. adamanteus* (Eastern Diamondback rattlesnake), *C. atrox* (Western Diamondback rattlesnake), *C. horridus* (Timber rattlesnake), and *A. p. piscivorus* (Eastern Cottonmouth) were obtained from a single adult snake from each snake species. Venoms were extracted from the snakes using disposable plastic cups covered with parafilm. The venoms were spun at 10,000× *g* at 4 °C, for 5 min using a Beckman Coulter Avanti 30 Centrifuge, filtered through a 0.45 µm MillexHV syringe filter unit (Millipore, Billerica, MA, USA), lyophilized, and stored at −80 °C until use.

### 4.2. Isolation of svCRiSPs

svCRiSPs were purified from lyophilized crude venoms using two sequential chromatographic procedures as previously described [10]. First, the venoms were separated by reverse phase HPLC using Higgins Analytical PROTO 300 C18 column (250 × 4.6 mm^2^, 5 µm) (Higgins Analytical, Inc., Mountain View, CA, USA), followed by cation-exchange on SP- 5PW Waters Protein-Pak™ column (7.5 × 75 mm^2^) (Waters Corp., Milford, MA, USA).

### 4.3. SDS-PAGE and N-Terminal Sequencing

Purified svCRiSPs (5 µg) were separated by a precast 4–12% (*w*/*v*) NuPAGE^®^ Novex Bis-Tris gels (Invitrogen™, Carlsbad, CA, USA) under non-reducing conditions. The gel was run at 100 V for 90 min with MES SDS running buffer using an XCell SureLock™ mini cell electrophoresis system (Invitrogen™) and a PowerPac Basic system (Bio-Rad Laboratories, CA, USA). SeeBlue^®^ Plus2 marker (Life Technologies™, Carlsbad, CA, USA) was used for estimated molecular weight. After electrophoresis, gel was visualized with SimplyBlue™ SafeStain (Life Technologies™).

Crotaline CRiSPs (4 µg) were subjected to SDS-PAGE and transferred to PVDF membrane (Millipore Immobilon, Carrigtwohill, Ireland) using a Transblot^®^ Semi-Dry Transfer Cell (Bio-Rad) at 100 mA for 90 min. The membrane was visualized with Coomassie brilliant blue R-250 staining (0.25% Coomassie Brilliant Blue R-250 in 40% methanol) for 5 min. Then, the protein bands were excised for N-terminal amino acid sequencing. The sequences of crotaline CRiSPs were determined using automated Edman degradation on a PPSQ-33B protein sequencer (SHIMADZU, Kyoto, Japan) according to the manufacturer’s instructions. The N-terminal amino acid sequences of svCRiSPs were identified and compared with other proteins using Basic Local Alignment Search Tool (BLAST—http://blast.ncbi.nlm.nih.gov/Blast.cgi, accessed on 1 April 2020).

### 4.4. Protein Identification of svCRiSPs by LC-MS/MS Analysis and Data Processing

To identify venom composition in each sample venoms and full amino acid sequences of svCRiSPs (Catrox-CRiSP from *C. atrox*, Css-CRiSP from *C. s. scutulatus*, Chor-CRiSP from *C. horridus*, Cada-CRiSP from *C. adamanteus*, and App-CRiSP from *A. p. piscivorus*), LC-MS/MS analysis were performed according to Suntravat et al. [10]. MaxQuant software (Version 1.6.2.6) was used to analyze the mass spectrometric (RAW) data. The match identifications of both protein and peptide-to-spectrum had a required false discovery rate (FDR) of 1%. The instrument was set as an Orbitrap, with all of the instrument settings remaining at default settings. The type of quantification was set to Label Free Quantification (LFQ) with the minimum LFQ count set to 2 and Fast LFQ was selected. Trypsin was selected as the specific digestion enzyme with an allowance of two missed cleavages. Under fixed modifications, cysteine carbamidomethylation was selected, and methionine oxidation and N-terminal acetylation were set as variable modifications. The “match-between-runs” and “requantify” features were enabled. The mass spectrometric data was performed against a family Viperidae database (Uniprot release 08/2020), which contained 1329 manually annotated (reviewed) sequences along with 28,008 computationally annotated (unreviewed) sequences. For protein quantification, the label minimum count ratio was set to two and the “match-between-runs” function was enabled under Advanced Identifications. After the analysis was completed, the software with the aid of Uniprot was used to identify CRiSP proteins and their charge-to-mass ratios along with the MS/MS scans. The relative abundance of the different venom protein families in each crude venom was expressed as the percent of the total venom proteins.

### 4.5. Multiple Sequence Alignment

The complete amino acid sequences of svCRiSPs derived from N-terminal amino acid sequencing and LC-MS/MS were identified with BLASTp program on the NCBI protein databases. Multiple sequence alignments and phylogenetic tree analysis of svCRiSPs were generated by a ClustalW method and a neighbor-joining method in the Lasergene MegAlign program (DNASTAR, Inc. Madison, WI, USA), respectively. The bootstrap analysis was performed using 1000 replications. Human CRiSP-3 was used to root the tree.

### 4.6. Vascular Permeability In Vivo (Miles Assay)

Groups of 5 BALB/C mice (18–21 g body weight, male and female) were intravenously injected with 0.1 mL of 1% Evans blue dye. Thereafter, 100 µL of svCRiSPs (170 ng/mouse), VEGF-A (170 ng/mouse, positive control), or normal saline (negative control) was injected subcutaneously into the dorsal area. Thirty minutes later, the mice were euthanized. The skins were dissected, photographed, and weighed. Evans blue dye was extracted by incubating the tissues in formamide for 48 h, at 55 °C and the optical density was measured at 620 nm. Values are expressed as fold increase relative to negative control.

### 4.7. Cell Cultures

Primary HDBEC and HDLEC cells were obtained from PromoCell (PromoCell GmbH, Heidelberg, Germany). HDBECs and HDLECs were grown in gelatin-coated 75 cm^2^ flasks under constant humidity (5% CO_2_, 37 °C) and supplemented with endothelial cell growth media MV and MV2 (PromoCell GmbH), respectively, containing 5% fetal bovine serum following the manufacturer’s instructions. All experiments were done at passages 3–6 to obtain consistent data.

### 4.8. Endothelial Permeability Assay

The endothelial permeability assay was carried out as described previously [10]. Briefly, HDLECs or HDBECs were grown on gelatin-coated transwell inserts (BD falcon) for 3 days to achieve confluence. After reaching confluence, cells were incubated with svCRiSPs at different concentrations (0.33, 0.67, and 1.3 µM) for 1 h. Then, 10 µL of FITC-BSA (10 mg/mL) was added into the insert in the top compartment. After 30 min, the sample was removed from the bottom compartment and FITC-BSA concentration of collected samples was measured using a fluoroskan Ascent FL microplate reader (Thermo Fisher Scientific, Waltham, MA, USA) with excitation 485 nm and emission 538 nm. The data are presented as fold change compared to control cells.

### 4.9. Reverse Phase Protein Array (RPPA)

Cells used for RPPA analysis were grown in six-well tissue culture plates. After the cells reached about 90–100% confluence, the culture media were aspirated, and the cells were cultivated in low serum (2%) media for 3 h. Then, cells were preincubated with 1 µM Css-CRiSP or App-CRiSP, or PBS for 30 min. The cells were washed two times with ice-cold PBS and then lysed on ice in RIPA buffer (Thermo Fisher Scientific) with 1% protease and phosphatase inhibitor cocktail (Sigma-Aldrich, St. Louis, MO, USA). After a 20-min incubation on ice, protein lysates were centrifuged for 10 min at 14,000 rpm and supernatants were collected. Protein concentration was quantified with a Pierce BCA protein assay kit (Thermo Fisher Scientific) and samples were adjusted to 1 mg/mL. The protein lysates were mixed with 4× SDS Sample Buffer (0.25 M Tris-HCl, pH 6.8 containing 8% SDS, 40% glycerol, and 10% 2-mercaptoethanol). The mixtures were incubated at 95 °C for 5 min and were sent to RPPA Core Facility at MD Anderson Cancer Center (Houston, TX, USA) for RPPA. The antibody list is available at https://www.mdanderson.org/research/research-resources/core-facilities/functional-proteomics-rppa-core/antibody-information-and-protocols.html, accessed on 30 August 2020.

### 4.10. Statistical Analysis

Unless otherwise noted, all results were obtained from at least two independent experiments done in triplicate. The data was presented as the means ± the standard deviation (SD). Data were calculated using Student’s *t*-test to study the possible differences between the experimental and control groups. *p* < 0.05 was accepted as statistically significant. RPPA experiments were carried out in duplicate. All signal intensities were normalized for protein loading and transformed to log2 values (Normlog2). Fold changes were calculated by comparing the signal obtained from the cell lysates of HDBECs and HDLECs treated with Css-CRiSP and App-CRiSP to control (untreated) samples. Differentially expressed total proteins and phosphorylated proteins were determined using Student’s *t* test (*p* < 0.05). Heat maps were created in Excel using XLSTAT add-on software version 2020.4.1. Proteins were ordered by the relative rank based on the normalized values.

## Figures and Tables

**Table 1:** Data obtained from LC-MS/MS analysis of tryptic digested peptides of svCRiSPs.

CRiSPs	Positions	Theo. MH+ [Da]	Sequenced Fragment
Css-CRiSP	30–36	856.48869	(R)KPEIQNK(I)
	37–45	1126.63675	(K)IVDLHNFLR(R)
	46–57	1333.68926	(R)RSVNPTASNMLK(M)
	58–74	2129.94978	(K)MEWYPEAAANAERWAYR(C)
	75–85	1343.61207	(R)CIESHSPRDSR(V)
	86–91	658.4134	(R)VLEGIK(C)
	92–104	1523.72326	(K)CGENIYMSPVPIK(W)
	105–117	1620.79175	(K)WTEIIHAWHGENK(D)
	121–142	2448.23466	(K)YGIGADPPNAVIGHYTQVVWYK(S)
	146–157	1284.57112	(R)IGCAAAYCPSSK(Y)
	158–175	2202.99871	(K)YSYFYVCQYCPAGNIIGK(T)
	176–181	680.36137	(K)TATPYK(S)
	182–212	3563.40587	(K)SGPPCGDCPSACDNGLCTNPCTKEDKYTNCK(S)
	213–238	3117.33151	(K)SLVQQAGCQDKQMQSDCPAICFCQNK(I)
	224–240	2128.92826	(K)QMQSDCPAICFCQNKII(-)
Catrox-CRiSP	30–45	1964.10761	(R)KPEIQNKIVDLHNFL(R)
	46–57	1333.68926	(R)RSVNPTASNMLK(M)
	58–74	2129.94978	(K)MEWYPEAAANAERWAYR(C)
	75–82	985.45199	(R)CIESHSPR(D)
	86–104	2091.09769	(R)VLGGIKCGENIYMSPVPIK(W)
	105–120	2009.99805	(K)WTEIIHAWHGENKNFK(Y)
	121–145	2796.42564	(K)YGIGAVPPNAVTGHFSQVVWYKSYR(I)
	146–175	3468.55199	(R)IGCAAAYCPSSKYSYFYVCQYCPAGNIIGK(T)
	176–204	3186.30535	(K)TATPYKSGPPCGDCPSACDNGLCTNPCTK(E)
	205–223	2272.03326	(K)EDKYTNCKSLVQQAGCQDK(Q)
	224–240	2128.92826	(K)QMQSDCPAICFCQNKII(-)
Cada-CRiSP	30–46	2060.17233	(R)KPEIQNKIVDLHNSLRR(S)
	47–57	1177.58815	(R)SVNPTASNMLK(M)
	58–74	2173.93961	(K)MEWYPEAADNAERWAYR(C)
	75–85	1329.59642	(R)CIDSHSPRDSR(V)
	83–104	2415.30644	(R)DSRVLGGIKCGENIYISPVPIK(W)
	105–117	1620.79175	(K)WTEIIHAWHGENK(N)
	121–142	2434.21901	(K)YGIGADPPNAVTGHFTQIVWYK(S)
	143–173	3700.53886	(K)SYHVGCAAAYCPSSEYSYFYVCQYCPAGNIR(G)
	174–181	865.47779	(R)GKTATPYK(S)
	182–212	3549.39022	(K)SGPPCGDCPSACDNGLCTNPCTKEDKYSNCK(S)
	213–240	3333.4789	(K)SLVQQAGCQDKQMQSDCSAICFCQNKII(-)
Chor-CRiSP	1–10	1180.51167	SVDFDSESPR(K)
	11–27	2061.11996	(R)KPEIQNEIVDLHNSLRR(S)
	28–38	1177.58815	(R)SVNPTASNMLK(M)
	39–55	2129.94978	(K)MEWYPEAAANAERWAYR(C)
	56–66	1343.61207	(R)CIESHSPRDSR(V)
	67–85	2163.11882	(R)VLEGIKCGENIYMSPVPIK(W)
	86–98	1606.7761	(K)WTEIIHGWHGENK(N)
	102–123	2438.21392	(K)YGIGAEPSNAVTGHFTQIVWYK(S)
	124–138	1676.75194	(K)SYRVGCAAAYCPSSK(Y)
	139–156	2246.01576	(K)YSYFYVCQYCPAGNIRGK(T)
	157–185	3186.30535	(K)TATPYKSGPPCGDCPSACDNGLCTNPCTK(E)
	186–204	2258.01761	(K)EDKYSNCKSLVQQAGCQDK(Q)
	205–221	2118.90753	(K)QMQSDCSAICFCQNKII(-)
App-CRiSP	30–36	856.48869	(R)KPEIQNK(I)
	37–45	1126.63675	(K)IVDLHNFL(R)
	46–57	1333.68926	(R)RSVNPTASNMLK(M)
	58–70	1553.66892	(K)MEWYPEAAANAER(W)
	75–82	985.45199	(R)CIESHSPR(D)
	92–104	1523.72326	(K)CGENIYMSPVPIK(W)
	105–117	1620.79175	(K)WTEIIHAWHGENK(N)
	121–142	2390.22918	(K)YGIGAVPPNAVTGHFSQVVWYK(S)
	146–157	1284.57112	(R)IGCAAAYCPSSK(Y)
	182–204	2524.96182	(K)SGPPCGDCPSACDNGLCTNPCTK(E)
	213–223	1233.58921	(K)SLVQQAGCQDK(Q)
	224–238	1902.76014	(K)QMQSDCPAICFCQNK(I)

## Data Availability

Not applicable.

## Supplementary Materials

The following are available online at https://www.mdpi.com/article/10.3390/toxins13090613/s1, Table S1: The relative composition of identified proteins from *C. s. scutulatus* venom. Table S2: The relative composition of identified proteins from *C. atrox* venom. Table S3: The relative composition of identified proteins from *C. adamanteus* venom. Table S4: The relative composition of identified proteins from *C. horridus* venom. Table S5: The relative composition of identified proteins from *A. p. piscivorus* venom. Table S6: The trypsin digested fragments of several partially overlapping and redundant peptides for svCRiSPs were sequenced by mass spectrometry. Table S7: Fold changes in HDBECs and HDLECs after treated with App-CRiSP and Css-CRiSP. Table S8: Quantification of fold changes in phosphorylation status of target proteins in treated cells over untreated cells as observed by RPPA.
